# Optimization of Gluten‐Free and Low‐GI Crispy Cookies From Agrowaste Flours Using Nutritional Sweetener and Lecithin: A Qualitative and Functional Assessment

**DOI:** 10.1155/ijfo/9974154

**Published:** 2026-06-12

**Authors:** Tiffany Arista Sutanto, Giovanni Aurelia Belinda, Christina Mumpuni Erawati, Ardhia Deasy Rosita Dewi, Maria Goretti Marianti Purwanto

**Affiliations:** ^1^ Fakultas Teknobiologi, Universitas Surabaya, Surabaya, Indonesia; ^2^ Center of Excellence for Food Products and Health Supplements for Degenerative Condition, Universitas Surabaya, Surabaya, Indonesia

## Abstract

Nowadays, food trends are heading towards gluten‐free and healthier food, including cookies, due to the rising prevalence of celiac disease and other gluten‐related disorders. One way to keep up with the trend is to use agrowaste flour. However, consumers’ acceptance of such cookies may be lower compared to that of cookies made with conventional wheat flour. To enhance both the sensory quality and nutritional value, food additives such as lecithin and low‐glycemic sweeteners can be incorporated. Lecithin improves the texture and appearance of crispy cookies, while low‐glycemic sweeteners help reduce the glycemic index and glycemic load without compromising consumer preference. The objective of this study was to evaluate and optimize the physical characteristics, sensory attributes, proximate composition, glycemic index, and glycemic load of crispy cookies formulated with durian and papaya seed flours, lecithin, and low‐glycemic sweeteners. The research was conducted in two stages: (1) improving texture by determining the optimal concentration of lecithin and (2) replacing refined sugar with an optimal combination of low‐glycemic sweeteners such as erythritol, xylitol, and stevia. The optimal concentration of lecithin was found to be 0.5%, while the most effective sweetener formulation based on the De Garmo effectiveness index consisted of 28.10% erythritol and 0.02% stevia. The resulting crispy cookies exhibited improved texture and appearance, a low glycemic index (32.55), and a low glycemic load (4.64), while maintaining good consumer acceptance. These findings highlight the potential of this formulation to be developed further as a functional cookie product suitable for health‐conscious consumers.

## 1. Introduction

Recent food trends have increasingly favored gluten‐free products, driven by the rising prevalence of gluten‐related disorders and growing public awareness [1]. This trend presents a valuable opportunity for countries like Indonesia, as wheat is the primary source of gluten in baked goods, which cannot be cultivated and must be imported. Reducing reliance on wheat through alternative, locally sourced ingredients could not only support health‐focused food innovations but also help decrease national wheat import volumes, which are 12.14 million tons in 2024 [2].

One way to keep up with the trend is to use agricultural waste (agrowaste) flours, such as durian seed and papaya seed flours (PSFs). These flours are naturally gluten‐free and possess considerable nutritional potential, particularly in terms of dietary fiber, which is typically low in conventional cookies (approximately 2.1%) [3]. In certain instances, specific agrowaste fractions may exhibit concentrated levels of bioactive compounds or dietary fiber that can surpass those found in the primary edible portions of the fruit [4]. Several studies have reported the potential of agrowaste‐derived flours—such as mango kernel, jackfruit seed, and banana peel flours—in improving the nutritional and functional quality of bakery products while reducing glycemic impact [5, 6].

Durian seed flour (DSF) and PSF represent underutilized by‐products with significant potential as wheat substitutes. Both durian and PSFs were expected to contribute not only to structural and textural improvements in gluten‐free cookies but also to enhance their functional and nutritional profile through bioactive and fiber‐rich components. These bioactive constituents are expected to enhance the functional quality and shelf life of the cookies while contributing to their potential health benefits. Previous analyses have shown that DSF contains approximately 70%–75% carbohydrates (mainly starch), 7%–9% protein, and 4%–5% crude fiber, with significant levels of resistant starch and polysaccharides that can contribute to lower postprandial glycemic responses [7]. Meanwhile, PSF contains around 25%–30% protein, 20%–25% dietary fiber, and bioactive phenolic compounds such as caffeic acid and p‐coumaric acid, which possess antioxidant and antimicrobial properties [8]. The combination of these nutrient‐dense agrowaste flours may improve both texture and nutritional quality by increasing fiber and protein content while reducing the glycemic index (GI) through slower carbohydrate digestion. These characteristics support the rationale for using durian and PSFs as functional, gluten‐free ingredients for developing healthier bakery products.

However, the incorporation of these agrowaste flours into bakery products presents significant challenges due to consumer’s acceptance for agrowaste cookies can be lower than using wheat flour. A previous study [9] formulated crispy cookies by partially replacing wheat flour with durian and PSFs [10]. The cookies exhibited noticeable changes in their sensory properties, including a darker color (resembling burnt cookies), a firmer texture, and a distinctive taste due to the PSF. Formulation with 50% wheat flour, 40% DSF, and 10% PSF showed the most promising results. However, sensory evaluations indicated moderate acceptance. Moreover, the physicochemical properties of the cookies were not investigated in the previous research, so further optimization is needed. Most existing research has prioritized the nutritional fortification of these flours, leaving a notable gap in the optimization required to achieve the “snap” and “crispiness” expected in commercial cookie products [5, 6, 9, 10].

This study is aimed at improving the formulation by implementing two key strategies: (1) improving the texture by finding the optimal concentration of lecithin and (2) replacing refined sugar with the optimal combination of low‐glycemic sweeteners such as erythritol, xylitol, and stevia. Lecithin, a natural emulsifier from phospholipids, can enhance dough homogeneity and soften cookie texture, which may improve overall texture and increase consumer acceptance [11]. The concentrations that will be used are 0%, 0.5%, 1.0%, and 1.5% of the batter weight [12]. To further improve taste and appearance, this study will incorporate cocoa powder into the cookie formulation, creating a chocolate‐flavored variant. The dark brown hue of cocoa powder complements the naturally dark color of cookies made with both seed flours, while the rich chocolate taste is expected to mask the pungent flavor of PSF, which is less desirable in the original flavor [9].

In 2022, Indonesia’s total sugar consumption reached 8.47 million tons—a more than 10% increase from the previous year [13]. High sugar intake is closely linked to the rising incidence of diabetes, which caused over 3.4 million deaths worldwide yearly. Replacing refined sugar with low‐glycemic sweeteners offers a healthier alternative that supports blood sugar control, reduces calorie intake, and contributes to diabetic‐friendly diets. Erythritol, a sugar alcohol found in fruits and fermented foods, provides about 70% of sucrose’s sweetness with negligible calories and minimal glycemic impact [14]. Xylitol, another sugar alcohol, offers similar sweetness with fewer calories (2.4 kcal/g) and additional health benefits, including antimicrobial and anti‐inflammatory properties [15]. Stevia (*Stevia rebaudiana*), a high‐intensity natural sweetener, is 150–300 times sweeter than sucrose, contains no calories, and has demonstrated antihyperglycemic, antioxidant, and antimicrobial activities [16].

Although these sweeteners can reduce GI and glycemic load (GL) [17], their use may alter texture and taste, potentially reducing consumer acceptance [18, 19]. Therefore, the purpose of this study is to evaluate and optimize the physical characteristics, sensory attributes, proximate composition, GI, and GL of crispy cookies made with durian and PSFs, lecithin, and low‐glycemic sweeteners. While previous studies have explored the individual nutritional profiles of durian and papaya seeds, research on their synergistic application in a composite flour system remains scarce. Specifically, there is a lack of evidence regarding how these flours interact within a gluten‐free matrix when stabilized by lecithin. Most existing studies focus on simple nutritional fortification, failing to address the gap between high‐fiber inclusion and the “crispy” structural requirements of a commercial cookie. Furthermore, the combined effect of a low GI sweetener system on the sensory perception and GL of such agrowaste‐based composites has not been systematically optimized. The final product is expected to be nutritious, gluten‐free, and environmentally sustainable, with a low GI and GL, making it appropriate and safe for consumption by individuals with diabetes. In addition, the chocolate flavor is anticipated to enhance sensory attributes, particularly taste and texture, which may improve overall consumer acceptance.

## 2. Materials and Methods

### 2.1. Materials

The raw materials used in this study included seeds of the California papaya variety (*Carica papaya* L.), which were locally sourced and processed into flour. DSF (*Durio zibethinus*) was obtained from Hasil Bumiku (Indonesia). Durian and papaya fruits were collected between September and November 2024, at the end of the dry season in Malang, Indonesia.

Other dry ingredients consisted of maize flour (Maizenaku, Indonesia), alkalized cocoa powder (Van Houten), vanilla powder (Polar Bear), and salt. The lipid source used was premium butter (Wijsman, Netherlands). For the sweetener system, powdered sugar (Mawar) was used for the control, while the low‐glycemic formulations utilized erythritol (Greenara), xylitol (NOW Foods, United States), and a high‐purity stevia extract (Sweet Story Stevia). Additional ingredients for texture and flavor included sliced almonds (Almonesia) and cheddar cheese (Kraft). Laboratory‐grade consumables included mineral water (Club) and aluminum foil (Klinpak).

All food ingredients were of food‐grade quality. All chemical reagents used in this study were of analytical grade and purchased from Sigma‐Aldrich.

### 2.2. Preparation of DSF

DSF was prepared following the method described in a previous study [9], with modifications. Durian seeds were sorted, washed, and boiled (90°C–100°C, 15 min). Then, they were drained, peeled, and thinly sliced. The boiled seeds were then drained, peeled, and thinly sliced before being soaked in a 5% NaCl solution for 2 h to remove the high concentration of surface mucilage (gum) and to prevent enzymatic browning. This step is essential for durian seeds to produce a stable, workable flour. After that, they were drained and dried using a cabinet dryer (Omron E5CSL, Jakarta, Indonesia) at 60°C for 23 h. The dried seeds were ground with a semiautomatic grinder (Fomac FCT‐Z200, Jakarta, Indonesia) for 5 min (high‐speed swing‐type, 25,000 RPM) and sieved through a 70‐mesh sieve (MBT Utama [ASTM Standard Test Sieve], Jakarta, Indonesia).

### 2.3. Preparation of PSF

PSF was prepared following a modified method from a previous study [9]. The seeds were sorted, washed, and drained to remove the pulp, sarcotesta, and other impurities. Then, they were dried in a cabinet dryer (Omron E5CSL, Jakarta, Indonesia) at 60°C for 23 h, ground with a semiautomatic grinder for 5 min, and sieved through a 70‐mesh sieve (MBT Utama [ASTM Standard Test Sieve], Jakarta, Indonesia). Preparation of papaya seeds did not require this treatment because they lack the specific mucilaginous structure found in durian seeds. Furthermore, skipping the saline soak for papaya seeds preserved the bioactive compounds and specific flavor notes required to complement the cocoa powder in the final formulation.

### 2.4. Preparation of the Chocolate Crispy Cookies With Lecithin

The formulation of chocolate crispy cookies is shown in Table 1. Cocoa powder was added at a fixed level of 6 g per 100 g total flour mixture (equivalent to 6% *w*/*w*), which was chosen based on preliminary trials to provide an optimal chocolate flavor and color without masking the sensory characteristics of the agrowaste flours.

**Table 1:** Formulation of chocolate crispy cookies with different concentrations of lecithin.

No.	Ingredients	Unit	Amount				
			K	P1	P2	P3	P4
1	Low‐protein wheat flour	g	10	0	0	0	0
2	Durian seed flour (DSF)	g	0	8	8	8	8
3	Papaya seed flour (PSF)	g	0	2	2	2	2
4	Lecithin	g	0	0	0.94	1.87	2.81
5	Cocoa powder	g	6	6	6	6	6
6	Butter	g	50	50	50	50	50
7	Powdered sugar	g	40	40	40	40	40
8	Egg whites	g	60	60	60	60	60
9	Vanilla powder	g	1	1	1	1	1
10	Maize flour	g	20	20	20	20	20
11	Almond slice (topping)	g	20	20	20	20	20
12	Grated cheese (topping)	g	10	10	10	10	10

*Note:* K = 100% wheat flour, without lecithin; P1 = 80% DSF, 20% PSF, and lecithin 0%; P2 = DSF, PSF, and lecithin 0.5%; P3 = DSF, PSF, and lecithin 1%; P4 = DSF, PSF, and lecithin 1.5%.

Initially, a small amount of butter was mixed with lecithin (0%, 0.5%, 1%, and 1.5%). Then, the mixture was combined with the remaining butter, powdered sugar, and vanilla powder. Then, egg whites were added until evenly mixed. Then, the sifted dry ingredients (cornstarch, cocoa powder, DSF, and PSF) were added. For the control formulation (K), only wheat flour was used, without lecithin or agrowaste flours.

The dough was transferred into a piping bag with a 0.5 cm cut tip and shaped into 20 spirals on a baking sheet lined with a baking mat and a silicone almond crispy mold. Each spiral was formed by rotating the piping bag five times, then thinned and flattened by pressing clockwise five times with a spoon. The prebacking cookies were assured to weigh around 5 g each with 2 mm thickness. Roasted almond slices and grated cheddar cheese were sprinkled on top before baking (electric convection oven, Modena BO‐2635, Surabaya, Indonesia; preheated at 120^°^C ± 2^°^C for 30 min, 40 ± 2 min baking time). After baking, the cookies were cooled at room temperature (25°C, 5 min) and subsequently packaged in airtight containers lined with baking paper and silica gel. The resulting baked cookies measured approximately 6 cm in diameter and 0.1–0.15 cm in thickness. Each formulation was prepared in triplicate batches to ensure reproducibility.

### 2.5. Preparation of the Chocolate Crispy Cookies With Low‐Glycemic Sweeteners

The formulation containing the optimal lecithin concentration was selected as the reference formula for preparing cookies with various combinations of low‐glycemic sweeteners, with the formulation shown in Table 2. The cocoa powder concentration (6% *w*/*w*) was maintained constant across all formulations to ensure comparable color and flavor intensity. The preparation procedure followed that described in Section 2.4.

**Table 2:** Chocolate crispy cookies formulation with a combination of low‐glycemic sweeteners.

No.	Ingredients	Unit	Amount				
			P2	P5	P6	P7	P8
1	Durian seed flour (DSF)	g	8	8	8	8	8
2	Papaya seed flour (PSF)	g	2	2	2	2	2
3	Lecithin	g	0.94	0.94	0.94	0.94	0.94
4	Cocoa powder	g	6	6	6	6	6
5	Butter	g	50	50	50	50	50
6	Powdered sugar	g	40	0	0	0	0
7	Erythritol	g	0	50	46.67	50	50
8	Xylitol	g	0	10	12	0	5
9	Stevia	g	0	0	0	0.032	0.028
10	Egg whites	g	60	60	60	60	60
11	Vanilla powder	g	1	1	1	1	1
12	Maize flour	g	20	20	20	20	20
13	Almond slice (topping)	g	20	20	20	20	20
14	Grated cheese (topping)	g	10	10	10	10	10

*Note:* P2 = 22.48% powdered sucrose; P5 = 28.10% erythritol and 5.62% xylitol; P6 = 26.23% erythritol and 6.74% xylitol; P7 = 28.10% erythritol and 0.02% stevia; P8 = 28.10% erythritol, 2.81% xylitol, and 0.016% stevia.

### 2.6. Sensory Analysis

The sensory evaluation was conducted following the methodology described in [9], with modifications to the procedure, hedonic scale, and evaluated parameters. The analysis comprised two sequential steps: First, assessed cookies formulated with varying lecithin concentrations, while the second, focused on cookies prepared with the optimal lecithin level combined with different low‐glycemic sweeteners.

A total of 30 untrained panelists (18–60 years old; 17 females and 13 males) participated in each session. Panelists were recruited from the University of Surabaya community (students and staff) based on specific criteria: aged 18–60; good health status; absence of allergies to gluten, dairy, or nuts; and a general preference for crispy and chocolate‐flavored cookies. While the age range was 18–60, 80% of participants fell within the 18–25 age bracket (university students). Demographic data, including gender and frequency of snack consumption, were recorded but did not show statistically significant effects on the hedonic scores (*p* > 0.05). Each sensory test was conducted in a sensory evaluation room under controlled lighting (6500 K, neutral white) and temperature (25^°^C ± 2^°^C). Samples were coded using three‐digit codes and presented to panelists in randomized and balanced order to minimize bias. Each panelist evaluated all samples in one session, and water was provided for palate cleansing between samples. All cookie samples complied with the microbial standards regulated in the Indonesian National Standard for biscuits (SNI 2973:2022).

Panelists completed a questionnaire containing instructions and a 6‐point hedonic scale ranging from 1 (*dislike very much*) to 6 (*like very much*) to evaluate color, aroma, texture, and taste. In the first phase, texture was further subdivided into four attributes: texture during biting, chewing, swallowing, and overall texture. Additionally, panelists rated hardness (when bitten), crispness (when chewed), and smoothness (when swallowed) on 6‐point scales defined as follows:

• Hardness (when bitten): 1 = *very soft* to 6 = *very hard*
• Crispness (chewed): 1 = *not very crispy* to 6 = *very crispy*
• Smoothness (swallowed): 1 = *very rough* to 6 = *very smooth*

In the second phase, texture was assessed without the hardness and smoothness subparameters. Instead, three additional attributes were included: sweetness (related to taste), aftertaste, and overall acceptance.

Each sensory session lasted approximately 45 min. Data were collected in triplicate sessions for reliability. Sensory evaluation results were analyzed to determine the optimal formulation using de Garmo’s effectiveness index [20].

### 2.7. Physical Analysis

Texture properties, including hardness and fracturability, were assessed using a texture analyzer (Agrosta V2, Agrosta, France) equipped with a conical probe. Cookie samples (thickness = 2 mm) were positioned between the probe and the base platform. The analysis was performed under the following settings: pull force of 1 g, high speed of 21 mm/s, low speed of 5 mm/s, and a stroke after contact of 1 mm. Hardness was determined as the maximum peak force recorded, while fracturability was defined as the force corresponding to the first positive peak in the force–distance curve.

Surface color (*L*∗, *a*∗, and *b*∗ values) of cookies formulated with low‐glycemic sweeteners was measured at the center using a color reader (Konica Minolta CR20, Minolta Co., Japan).

Water activity of lecithin‐containing cookies was measured using a water activity meter (Rotronic HP‐23‐AW Set14, Rotronic AG, Sweden). Samples were filled to the indicated line in the sample container. Then, the container was secured in the sample holder. Readings were taken after stabilization (approximately 4–5 min).

All measurements were conducted in triplicate using different cookie samples for each replicate.

### 2.8. Proximate Analysis

The proximate analysis was determined on cookies with the optimal formulation on both steps (P2 with 0.5% lecithin and P7 with 28.10% erythritol and 0.02% stevia). The proximate analysis parameters for both cookies include moisture, ash, fat, protein, crude fiber [21], carbohydrate (by difference), and dietary fiber [22].

### 2.9. Determination of GI and Load

The determination of GI and GL was only conducted for the cookie formulation with the optimal lecithin concentration (P2, 0.5% lecithin) and low‐glycemic sweetener combination (P7, 28.10% erythritol and 0.02% stevia). The study involved 10 healthy adult subjects who met the inclusion criteria: aged 20–25, normal fasting blood glucose (70–100 mg/dL), BMI 18.5–25.0, no complications, no metabolic disorders, and no diet regimen in the previous 3 months. Exclusion criteria include use of medications, smoking, alcohol consumption, pregnancy or lactation, and history of chronic or metabolic diseases (e.g., diabetes, liver, and kidney disorders).

The protocol followed guidelines from the Indonesian Food and Drug Control Agency [23] and was approved by the Institutional Ethical Committee of the University of Surabaya (No. 485/KE/I/2025). After 10–12 h of fasting, blood glucose was measured by finger‐prick capillary blood sampling. Subjects consumed 50 g of glucose in 200 mL of water (reference food), and glucose levels were measured at 0, 30, 60, 90, and 120 min. After a 4‐day washout period, the procedure was repeated using the cookie sample (P2 or P7), containing 25 g of available carbohydrates. The portion size of cookies equivalent to 25 g of available carbohydrates was calculated based on the carbohydrate content obtained from proximate analysis, corresponding to approximately 51.9 g of cookies for P2 and 52.6 g for P7.

GI was calculated by comparing the area under the curve (AUC) of the test food to that of the reference food. The AUC, GI, and GL were calculated using the following formula:

AUC=Δ30t2+Δ6090120t+Δ3060−Δt2+Δt+Δ6090−Δt2+Δt+Δ90120−Δt2,

Glycemic index GI=AUC of test food×2AUC of reference food×100,

Glycemic load GL=IG×total carbohydrate in one serving100.

### 2.10. Statistical Analysis

Data normality and homogeneity were assessed using Shapiro–Wilk and Levene’s tests, respectively. Organoleptic data were analyzed using the Kruskal–Wallis test, followed by Mann–Whitney (first development step) or Dunn’s post hoc test (second development step). Physical characteristics were analyzed using one‐way ANOVA with DMRT post hoc test or independent *t*‐test (second step). Proximate analysis was also performed using an independent *t*‐test. All tests used a significance level (*α*) of 5% and were carried out using IBM SPSS Statistics Version 29.0. (IBM Corp., Armonk, New York, United States).

## 3. Results and Discussion

### 3.1. The Development of Cookies With Lecithin

#### 3.1.1. Physical Analysis

Cookies made with DSF, PSF, and lecithin exhibited significantly higher hardness and fracturability compared to the control sample made with 100% wheat flour (Table 3). These findings suggest that incorporating agrowaste flours increases the force required to break the cookies, consistent with the results reported in the previous study [24]. This effect is likely due to differences in textural components such as amylose, carbohydrates, fat, dietary fiber, and protein content. Higher levels of lecithin increased batter viscosity as a result of its emulsifying properties, which reduced air incorporation and led to denser, harder cookies.

**Table 3:** Physical properties of crispy cookies with variation of lecithin concentration.

Formulation	K	P1	P2	P3	P4
Hardness (cN)	59 ± 17^a^	151 ± 6^b^	165 ± 11^b^	242 ± 12^c^	274 ± 9^d^
Fracturability (cN)	53 ± 13^a^	107 ± 16^b^	129 ± 18^b^	206 ± 36^c^	223 ± 42^c^
Water activity (Aw)	0.30 ± 0.02^a^	0.38 ± 0.04^b^	0.27 ± 0.01^a^	0.30 ± 0.02^a^	0.33 ± 0.07^ab^

*Note:* Values refer to the arithmetic mean ± standard deviation. Different letters in the same row indicate significant differences between formulations based on the DMRT post hoc test (*p* < 0.05).

The lowest water activity (Aw) was observed in sample P2 (DSF, PSF, and 0.5% lecithin). Formulations containing lecithin (P2 and P3) generally had lower Aw values than the wheat‐based formulation (K), which can be attributed to lecithin’s amphiphilic nature—its hydrophilic head binds water molecules, thereby reducing the amount of free water [25]. As the amount of free water decreases, the Aw value correspondingly declines. However, at higher lecithin concentrations (e.g., P4), the Aw value increased beyond that of the control, which could potentially compromise shelf life and cause undesirable textural changes, such as sogginess.

#### 3.1.2. Organoleptic Analysis

The addition of lecithin significantly affected consumer preference of the color (Table 4). Formulations P2–P4 (0.5%–1.5% lecithin) differed significantly from K and P1 (without lecithin), though no differences were observed in P2–P4, likely due to the dominant dark color of cocoa powder. The Maillard reaction, which produces melanoidin compounds, may also have contributed to the browning effect [26]. Cookies containing lecithin appeared smoother, brighter, and more uniform, thereby enhancing their visual appeal.

**Table 4:** Organoleptic properties of crispy cookies with various lecithin concentrations.

Parameter	Formulation				
	K	P1	P2	P3	P4
Color	4.00 ± 1.15^a^	3.96 ± 1.37^a^	4.96 ± 1.05^b^	5.07 ± 0.89^b^	4.91 ± 0.90^b^
Aroma	4.67 ± 1.18^ab^	4.41 ± 1.12^a^	4.89 ± 1.18^b^	5.07 ± 0.95^b^	4.69 ± 1.13^ab^
Texture when bitten	4.39 ± 1.38^a^	4.48 ± 1.33^a^	4.78 ± 0.86^a^	4.81 ± 1.15^a^	4.80 ± 1.20^a^
Texture when chewed	4.28 ± 1.41^a^	4.44 ± 1.11^a^	4.70 ± 1.00^a^	4.91 ± 1.07^b^	4.87 ± 1.17^b^
Texture when swallowed	4.44 ± 1.24^ab^	4.30 ± 1.13^a^	4.63 ± 1.00^ab^	4.78 ± 1.16^bc^	5.06 ± 0.81^c^
Texture (overall)	4.11 ± 1.48^a^	4.22 ± 1.24^a^	4.89 ± 1.02^b^	5.00 ± 0.91^b^	4.87 ± 1.03^b^
Taste	4.44 ± 1.24^ab^	4.24 ± 1.18^a^	5.02 ± 1.00^c^	4.98 ± 1.18^c^	4.87 ± 1.01^bc^

*Note:* Values refer to the arithmetic mean ± standard deviation. Different letters in the same row indicate significant differences between formulations based on the Mann–Whitney post hoc test (*p* < 0.05). K = 100% wheat flour, 0% lecithin; P1 = DSF, PSF, and lecithin 0%; P2 = DSF, PSF, and lecithin 0.5%; P3 = DSF, PSF, and lecithin 1%; P4 = DSF, PSF, and lecithin 1.5%. Scale 1 = *dislike very much*, 2 = *dislike moderately*, 3 = *slightly dislike*, 4 = *slightly like*, 5 = *like moderately*, and 6 = *like very much*.

Aroma preference was also improved in lecithin‐containing formulations (P2–P4) (Table 4). Lecithin’s amphiphilic nature facilitates better homogenization of both hydrophilic and lipophilic aroma compounds [25], which likely enhanced aroma distribution. The characteristic scent of lecithin itself was not prominent, possibly masked by stronger aromatic ingredients such as cocoa, butter, and cheese.

Texture preference during biting showed no statistically significant differences; however, the scores for P2–P4 were higher than those for K and P1 (Table 4). During chewing, K was described as sticky, whereas P1 was perceived as gritty, potentially due to the coarse particle morphology of PSF, as observed under SEM [27]. P3 (1% lecithin) received the highest preference score, being characterized by its crispy and nongritty texture. A notable divergence was observed between instrumental hardness values and sensory texture scores. While instrumental analysis indicated that higher lecithin concentrations (P4) resulted in increased peak force (hardness), sensory panelists rated these cookies as having a more desirable “softness” and “crispiness.” This phenomenon can be attributed to lecithin’s role as an emulsifier, which facilitates the formation of a more uniform and aerated microstructure. While the machine measures the absolute force required for initial fracture, the panelists perceive the “ease of mastication” and “shatter‐rate.” In the absence of lecithin (P1/K), the lack of emulsification likely led to a denser, more compacted matrix that felt “tougher” or more “unpleasant” to the human palate, despite having different instrumental force profiles.

Overall, lecithin inclusion showed a positive trend in enhancing textural attributes; however, the differences in “texture when bitten” were not statistically significant (*p* > 0.05) as shown in Table 5. Nevertheless, the overall acceptability scores suggest that lecithin contributes to a more cohesive and commercially viable gluten‐free structure. Taste preference also improved with the addition of lecithin. Its amphiphilic and zwitterionic properties promote emulsification and flavor uniformity [25], likely resulting in a more balanced and palatable taste profile.

**Table 5:** Hardness level (when bitten), crispness (when chewed), and smoothness level (when swallowed) of crispy cookies with various lecithin concentrations.

Parameter	Formulation				
	K	P1	P2	P3	P4
Hardness	3.46 ± 1.19^b^	4.04 ± 0.89^c^	2.87 ± 1.03^a^	2.72 ± 1.20^a^	2.59 ± 1.22^a^
Crispness	5.30 ± 0.86^b^	5.24 ± 0.78^b^	4.52 ± 1.04^a^	4.39 ± 1.05^a^	4.31 ± 0.99^a^
Smoothness	3.67 ± 1.17^a^	3.41 ± 1.07^a^	4.35 ± 1.20^b^	4.80 ± 1.05^bc^	4.94 ± 1.00^c^

*Note:* Values refer to arithmetic mean ± standard deviation. Different superscript letters in the same row indicate significant differences between formulations based on DMRT post hoc test (*p* < 0.05). Hardness level: Scale 1 = *very soft*; 2 = *soft*; 3 = *slightly soft*; 4 = *slightly hard*; 5 = *hard*; 6 = *very hard*. Crispness level: Scale 1 = *not very crispy*; 2 = *not crispy*; 3 = *slightly not crispy*; 4 = *slightly crispy*; 5 = *crispy*; 6 = *very crispy*. Smoothness level: Scale 1 = *very rough*; 2 = *rough*; 3 = *slightly rough*; 4 = *slightly smooth*; 5 = *smooth*; 6 = *very crispy*.

#### 3.1.3. Determination of the Optimal Formulation

The optimal formulation of crispy cookies with lecithin was determined by the de Garmo effectiveness index [20] (Table 6). The parameters considered in the optimization were sensory attributes, including color, aroma, overall texture, and taste.

**Table 6:** De Garmo effectiveness index for cookies with various concentrations of lecithin.

Parameter	Value	Formulation									
		K		P1		P2		P3		P4	
		NE	Nh	NE	Nh	NE	Nh	NE	Nh	NE	Nh
Color	0.10	0.00	0.00	0.000	0.00	1.00	0.10	1.00	0.10	1.00	0.10
Aroma	0.20	0.47	0.10	0.000	0.00	1.00	0.20	1.00	0.20	0.47	0.10
Texture (overall)	0.35	0.00	0.00	0.000	0.00	1.00	0.35	1.00	0.35	1.00	0.35
Taste	0.35	0.26	0.09	0.000	0.00	1.00	0.35	1.00	0.35	0.83	0.29
Total	1.00		0.19		0.00		1.00^a^		1.00		0.84

*Note:* NE = effectiveness value; Nh = productivity value; K = 100% wheat flour, 0% lecithin; P1 = DSF, PSF, and lecithin 0%; P2 = DSF, PSF, and lecithin 0.5%; P3 = DSF, PSF, and lecithin 1%; P4 = DSF, PSF, and lecithin 1.5%.

^a^The optimal formulation.

Parameter values were determined based on the research objective—to optimize the texture of crispy cookies—and consumer input from the ranking test, which indicated that taste and texture were equally important (data not shown). Accordingly, taste and texture were assigned equal weights (0.350). The optimal formulation was identified as the one with the highest Nh (productivity value). Both P2 and P3 achieved identical productivity values; however, P2 (0.5% lecithin) was selected as the optimum formulation due to its effectiveness and cost‐efficiency. Notably, the addition of only 0.5% lecithin produced sensory results comparable to P3, while requiring less lecithin. These findings suggest that P2 provides the best balance between product quality and economic feasibility for crispy cookie production. The similarity in textural performance between 0.5% and 1% lecithin (P2 and P3) suggests a possible saturation effect, where available hydrophilic and lipophilic interfaces in the dough become fully occupied at approximately 0.5% lecithin. Beyond this concentration, additional lecithin molecules may aggregate rather than contribute to further emulsification, limiting their ability to enhance air incorporation and structural homogeneity. Furthermore, lecithin may interact with proteins through hydrogen bonding and with dietary fibers through hydrophobic association, strengthening the dough matrix and increasing hardness up to the saturation point. Once this limit is reached, the system becomes more viscous and compact, but without proportional improvement in texture, explaining why 0.5% lecithin yielded similar or even superior results compared to 1%.

### 3.2. The Development of Cookies With Combined Low‐Glycemic Sweeteners

#### 3.2.1. Organoleptic Analysis

The combination of low‐glycemic sweeteners significantly influenced color preference (Table 7). Crispy cookies in formulation P2 received the lowest score, whereas P7 and P8 were rated the highest. The reddish‐brown hue of P2, attributed to powdered sugar, may have been less appealing to consumers. This coloration likely resulted from intensified Maillard reactions and caramelization, which produce brown pigments [26].

**Table 7:** Organoleptic properties of crispy cookies with a combination of low‐glycemic sweeteners.

Parameter	Formulation				
	P2	P5	P6	P7	P8
Color	4.13 ± 1.33^a^	4.60 ± 0.72^ab^	4.69 ± 0.82^ab^	4.89 ± 0.98^b^	4.89 ± 0.83^b^
Aroma	4.56 ± 0.89^a^	4.98 ± 0.75^a^	4.78 ± 0.80^a^	4.71 ± 1.10^a^	4.89 ± 1.01^a^
Crispness	5.56 ± 0.69^d^	3.98 ± 0.97^a^	4.38 ± 1.09^ab^	5.20 ± 0.87^cd^	4.76 ± 0.80^bc^
Texture (overall)	5.13 ± 0.89^c^	4.16 ± 0.88^a^	4.31 ± 1.08^ab^	5.00 ± 0.98^c^	4.93 ± 0.72^bc^
Sweetness	4.51 ± 1.04^ab^	4.56 ± 0.94^ab^	4.27 ± 1.03^a^	4.89 ± 1.07^b^	5.7 ± 0.75^b^
Taste	4.51 ± 1.24^ab^	4.51 ± 0.90^ab^	4.11 ± 1.05^a^	4.98 ± 1.06^b^	5.022 ± 0.75^b^
Aftertaste	4.29 ± 1.25^ab^	4.09 ± 0.10^a^	3.89 ± 1.07^a^	4.87 ± 1.04^b^	4.91 ± 0.923
Overall acceptance	4.62 ± 1.01^ab^	4.38 ± 0.75^a^	4.18 ± 0.91^a^	5.04 ± 0.95^b^	5.02 ± 0.75^b^

*Note:* Values refer to the arithmetic mean ± standard deviation. Different letters in the same row indicate significant differences between formulations based on the Dunn post hoc test (*p* < 0.05). P2 = 22.48% sucrose; P5 = 28.10% erythritol and 5.62% xylitol; P6 = 26.23% erythritol and 6.74% xylitol; P7 = 28.10% erythritol and 0.02% stevia; P8 = 28.10% erythritol, 2.81% xylitol, and 0.016% stevia.

Low‐glycemic sweeteners did not cause significant differences in aroma (Table 7). The cookies’ aroma mainly originated from chemical reactions such as the Maillard reaction [28]. However, cookies sweetened with erythritol, xylitol, or stevia (P5–P8) exhibited milder aromas than P2, likely due to their lower content of reducing sugars. Despite this, their aroma was generally well accepted, indicating no negative impact on consumer preference.

Crispness and texture were significantly affected by the type of sweetener used. P2, prepared with powdered sugar, underwent Maillard reactions, caramelization, and uniform sugar melting, leading to well‐developed porosity and crispness [28]. In contrast, erythritol and xylitol—which recrystallize rapidly and do not participate in the Maillard reaction—produced denser, less porous cookies with harder textures, consistent with previous findings [17].

Sweetness, taste, and aftertaste scores also varied significantly (Table 7). P2 had the lowest sweetness score, while P8 achieved the highest across all three parameters. P6 received the lowest taste and aftertaste scores, likely due to the fermented note associated with xylitol, which may be off‐putting to consumers [29]. Conversely, P7 and P8 were preferred, possibly due to erythritol’s cooling effect, which can enhance flavor perception and mask bitterness [30].

Overall acceptance differed significantly among the samples, with P6 being the least favored and P7 receiving the highest preference score (Table 7).

#### 3.2.2. Determination of Optimal Formulation

The combination of low‐glycemic sweeteners significantly influenced the sensory attributes of the crispy cookies. Among all formulations, P7 (28.10% erythritol and 0.02% stevia) exhibited the highest overall productivity value (Table 8). This formulation achieved the greatest effectiveness and productivity scores across all evaluated parameters, except for crispness, which ranked second. Despite its slightly lower crispness score, P7 remained highly acceptable to panelists, suggesting that it provides a favorable balance of taste, texture, and overall consumer appeal. Therefore, P7 can be considered the optimal formulation, offering both functional and sensory advantages for the development of low‐glycemic crispy cookies.

**Table 8:** De Garmo effectiveness index for cookies with low‐glycemic sweeteners (second development step).

Parameter	Value	Formulation									
		P2		P5	P6		P7		P8		
		NE	Nh	NE	Nh	NE	Nh	NE	Nh	NE	Nh
Crispness	0.25	1.00	0.25	0.00	0.00	0.25	0.06	0.78	0.19	0.49	0.12
Texture	0.21	1.00	0.21	0.00	0.00	0.17	0.04	1.00	0.21	0.85	0.18
Sweetmess	0.18	0.37	0.07	0.37	0.07	0.00	0.00	1.00	0.18	1.00	0.18
Taste	0.14	0.45	0.06	0.45	0.06	0.00	0.00	1.00	0.14	1.00	0.14
Aftertaste	0.11	0.33	0.04	0.00	0.00	0.00	0.00	1.00	0.11	1.00	0.11
Color	0.07	0.00	0.00	0.68	0.05	0.68	0.05	1.00	0.07	1.00	0.07
Overall acceptance	0.04	0.46	0.02	0.00	0.00	0.00	0.00	1.00	0.04	1.00	0.04
Total	1.00	3.61	0.65	1.50	0.18	1.10	0.15	6.78	0.94^*^	6.35	0.84

*Note:* NE = effectiveness value; Nh = productivity value. P2 = 22.48% sugar; P5 = 28.10% erythritol and 5.62% xylitol; P6 = 26.23% erythritol and 6.74% xylitol; P7 = 28.10% erythritol and 0.02% stevia; P8 = 28.10% erythritol, 2.81% xylitol, and 0.016% stevia.

^*^The optimal formulation.

#### 3.2.3. Physical Analysis

Objectively, the addition of low‐glycemic sweeteners did not significantly affect the color parameters of the cookies (Table 9). Both formulations showed low *L*∗ values, indicating a darker color, while the positive *a*∗ and *b*∗ values reflected reddish and yellowish hues. These color characteristics likely resulted from the natural pigments present in cocoa powder, PSF, and DSF, as well as from Maillard and caramelization reactions [31]. Although the difference was not statistically significant, P7 received slightly higher color preference scores than the control (P2), with panelists favoring a dark brown appearance featuring subtle reddish‐yellow tones.

**Table 9:** Physical properties for cookies with a combination of sweeteners.

Parameters	P2	P7
*L*∗ (lightness)	38.90 ± 0.20^a^	39.00 ± 0.44^a^
*a*∗ (redness)	6.03 ± 0.45^a^	5.37 ± 0.45^a^
*b*∗ (yellowness)	6.37 ± 0.45^a^	5.30 ± 0.56^a^
Hardness (cN)	161.67 ± 26.76^a^	243.33 ± 17.47^b^
Fracturability (cN)	132.67 ± 30.29^a^	216.00 ± 26.00^b^

*Note:* Values refer to the arithmetic mean ± standard deviation. Different letters in the same row indicate significant differences between formulations based on the DMRT post hoc test (*p* < 0.05).

The use of erythritol and stevia increased hardness and fracturability, consistent with previous findings in cookies made with ashitaba (*Angelica keiskei*) flour and erythritol [32] and those made with black glutinous rice flour [17]. The rapid recrystallization of sugar alcohols compared to sucrose likely contributed to a denser structure and reduced porosity, resulting in a firmer, less crispy texture.

Our observations that erythritol‐ and xylitol‐containing cookies (P5–P8) tended to be denser and harder than the sucrose control (P2) are consistent with prior reports on the physical behavior of polyols in baked systems [33]. Unlike sucrose, sugar alcohols such as erythritol and xylitol crystallize and recrystallize rapidly during cooling, which promotes the formation of fine crystals and reduces the continuity of the amorphous sugar phase. This recrystallization increases solid packing and reduces pore formation during baking, producing a denser crumb structure and higher hardness/fracturability. In addition, erythritol and xylitol do not participate in Maillard reactions (or do so negligibly), so they do not contribute to the uniform melting and glassy matrix formation that sucrose often provides; this difference also reduces the formation of continuous gas‐holding films and porosity, further explaining the reduced crispness.

Erythritol’s characteristic cooling effect may also modulate perceived sweetness and aftertaste, improving overall liking in formulations where crystallization does not produce an undesirable grainy mouthfeel. Xylitol has been reported to impart a fermented or off‐note in some bakery contexts, which aligns with the lower taste/aftertaste scores for P6. The combined sensory outcome therefore depends on a balance between recrystallization kinetics (affecting microstructure), hygroscopicity (influencing moisture retention and shelf behavior), and flavor interactions. Our results (higher hardness and fracturability in P7 relative to P2) fit this multifactor explanation and echo findings from other studies on polyol‐sweetened baked goods.

#### 3.2.4. Proximate Analysis

The use of low‐glycemic sweeteners such as erythritol and stevia (in P7) did not significantly affect the protein, fat, ash, or crude fiber contents of the cookies (Table 10), consistent with the nutritional profile of stevia, which contains negligible macronutrients. Although mineral content from unrefined sweeteners may influence ash levels [34], no significant changes were observed. Both P2 and P7 met the Indonesian National Standard (SNI 2973:2022) minimum protein requirement of 4.5%.

**Table 10:** Chemical properties of cookies with a combination of low‐glycemic sweeteners.

Composition	P2	P7
Protein (%)	13.63 ± 0.14^a^	13.48 ± 0.25^a^
Fat (%)	35.11 ± 0.04^a^	34.94 ± 0.37^a^
Moisture (%)	1.64 ± 0.08^a^	2.68 ± 0.02^b^
Ash (%)	1.43 ± 0.00^a^	1.41 ± 0.01^a^
Carbohydrate (%)	48.19 ± 0.24^b^	47.49 ± 0.36^a^
Crude fiber (%)	4.34 ± 0.07^a^	4.19 ± 0.10^a^
Dietary fiber (%)	10.74	10.48

*Note:* Values refer to the arithmetic mean ± standard deviation. Different letters in the same row indicate significant differences between formulations based on a *t*‐test (*p* < 0.05). P2 = cookies with 22.48% sucrose as sweetener; P7 = cookies with 28.10% erythritol and 0.02% stevia as sweetener.

The low‐glycemic sweeteners, however, affected the moisture content of the cookies. P7 exhibited significantly higher moisture content than P2 (Table 10), likely due to erythritol’s rapid recrystallization and the formation of a denser matrix that retains moisture despite its lower hygroscopicity [17, 28, 35]. In contrast, powdered sugar in P2 promoted better water evaporation through pore formation during baking. Both samples complied with the maximum moisture limit of 5% set by the Indonesian National Standard.

The energy and carbohydrate contents of P7 were lower than those of P2 (Table 10), owing to the use of erythritol and stevia, which provide 0.24 and 0 kcal/g, respectively, compared to sucrose’s 4 kcal/g [36, 37]. Erythritol, being a sugar alcohol, is also poorly metabolized, further reducing available carbohydrates.

Both cookie formulations contained approximately 10% dietary fiber (Table 10), attributed to the high fiber content of DSF and PSF, although these values may vary depending on the specific variety and processing conditions.

#### 3.2.5. GI and GL

**Figure 1:**
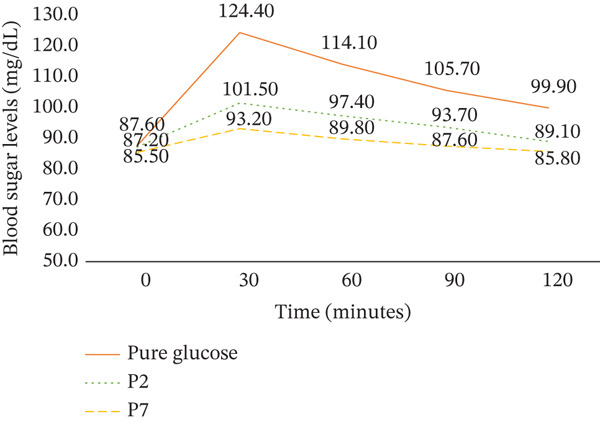
Blood sugar levels of crispy cookies with a combination of low‐glycemic sweeteners. P2 = cookies with 22.48% sucrose as sweetener; P7 = cookies with 28.10% erythritol and 0.02% stevia as sweetener.

Blood glucose measurements (Figure 1) represent the mean values obtained from 10 subjects. All formulations showed peak glucose levels 30 min after consumption, followed by a gradual decline. Among the samples, P7 produced the lowest glycemic response compared to both the glucose reference and the control (P2) (Table 11). This result aligns with its sweetener composition—erythritol and stevia—which both have a GI of 0, in contrast to sucrose in P2 (GI = 65), classified as a medium‐GI carbohydrate [38–40].

**Table 11:** Chemical properties of cookies with the optimal concentration of lecithin.

Parameters	Formulation	
	P2	P7
Glycemic index (GI)	72.99	32.55
GI category	High	Low
Glycemic load^a^	10.55	4.64
GL category	Medium	Low

*Note:* P2 = cookies with 22.48% sucrose as sweetener; P7 = cookies with 28.10% erythritol and 0.02% stevia as sweetener.

^a^Calculation based on serving size 30 g.

GL was also calculated based on a standard 30 g serving size [41]. P2 exhibited a medium GL, while P7 demonstrated a low GL (Table 11). Diets with low GI and GL values have been associated with improved glycemic control and reduced postprandial glucose spikes [42–44].

Accordingly, P7 presents a promising functional snack option for individuals who require blood glucose management. A 30 g serving provides approximately 10 g of erythritol and 6.4 mg of stevia, making it suitable for individuals aged over 10 years and weighing at least 20 kg, thereby minimizing the potential risk of gastrointestinal discomfort in younger children.

From a commercial standpoint, P7 demonstrates strong market potential in Indonesia, where low‐GI cookie products remain limited. Growing health awareness and increasing consumer demand for diabetic‐friendly foods further enhance its prospects for successful market adoption.

Despite the significant findings regarding the metabolic impact of the optimized cookie formulations, this study has certain limitations that should be acknowledged. Primarily, the GI and GL determinations were conducted with a relatively small sample size (*n* = 10). While this cohort size aligns with the minimum requirements set by international standards for preliminary glycemic testing (ISO 26642:2010), it may not fully account for the high interindividual variability in postprandial glucose responses across broader, more diverse populations. Furthermore, the sensory evaluation utilized a semitrained panel of 30 individuals, which, while sufficient for hedonic preference testing, may not capture the long‐term consumer acceptance or the “satiety” effects of agrowaste‐based snacks in a real‐world setting. Future research should involve larger, multicenter clinical trials and longitudinal sensory studies to validate the health benefits and market viability of these functional cookies.

## 4. Conclusions

This study successfully demonstrated that a combination of durian and PSFs can serve as a viable gluten‐free base for functional crispy cookies. By optimizing the concentration of lecithin (0.5%) and using a binary sweetener system (28.10% erythritol and 0.02% stevia), the research overcame the common textural and sensory hurdles associated with agrowaste‐based products. The resulting formulation (P7) not only met consumer preference for sweetness and crispiness but also achieved a clinically significant low GI (32.55) and low GL (4.64). These findings provide a technical framework for the food industry to valorize agrowaste into high‐value, diabetic‐friendly snacks. Future research should focus on evaluating the shelf life stability of these formulations, particularly the lipid oxidation of seed‐based fats, and product diversification, such as fortification with additional functional ingredients, could further enhance its commercial viability and market acceptance.

**Nomenclature**
Aw: water activity
ANOVA: analysis of variance
DSF: durian seed flour
GI: glycemic index
GL: glycemic load
PSF: papaya seed flour
SEM: scanning electron microscopy
T1–T4: treatment/formulation groups (lecithin optimization)
P1–P7: panel/formulation groups (sweetener optimization)

## Author Contributions

Tiffany Arista Sutanto and Giovanni Aurelia Belinda, B.Sc. in Biotechnology, performed most of the experiments and contributed to writing the paper, particularly the Methodology and Results and Discussion sections. Maria Goretti Marianti Purwanto, Ph.D. in Bioanalytical Chemistry, conceived and designed the experiments; analyzed and interpreted the data; secured the main research funding (covering reagents, materials, analytical tools, and data); wrote parts of the Introduction, Results and Discussion, and Conclusion sections; and served as the corresponding author. Ardhia Deasy Rosita Dewi, Ph.D. student in Food Technology analyzed and interpreted the data and contributed reagents and materials as part of the funded research team. Christina Mumpuni Erawati, Ph.D. student in Food Technology, analyzed and interpreted the data and contributed to manuscript writing, particularly in the Results and Discussion section. Tiffany Arista Sutanto and Giovanni Aurelia Belinda have contributed to the work equally and should be regarded as co‐first authors.

## Funding

The study was funded by Kementerian Riset Teknologi Dan Pendidikan Tinggi Republik Indonesia (10.13039/501100010447).

## Conflicts of Interest

The authors declare no conflicts of interest.

## Data Availability

The data that support the findings of this study are available from the corresponding author upon reasonable request.
